# Shengxian decoction protects against chronic heart failure in a rat model via energy regulation mechanisms

**DOI:** 10.1186/s12906-023-04035-3

**Published:** 2023-06-17

**Authors:** Ze-Qi Yang, Yang-Yang Han, Fan Gao, Jia-Ye Tian, Ran Bai, Qiu-Hong Guo, Xing-Chao Liu

**Affiliations:** Hebei University of Chinese Medicine, Xinshi South Road No 326, Qiaoxi District, Shijiazhuang, 050091 Hebei China

**Keywords:** Chronic heart failure, Shengxian decoction, Energy metabolism, Cardiac function, Myocardial structure

## Abstract

**Background:**

Chronic heart failure (CHF) is actually a disease caused by an imbalanced energy metabolism between myocardial energy demand and supply, ultimately resulting in abnormal myocardial cell structure and function. Energy metabolism imbalance plays an important role in the pathological process of chronic heart failure (CHF). Improving myocardial energy metabolism is a new strategy for the treatment of CHF. Shengxian decoction (SXT), a well-known traditional Chinese medicine (TCM) formula, has good therapeutic effects on the cardiovascular system. However, the effects of SXT on the energy metabolism of CHF is unclear. In this study, we probed the regulating effects of SXT on energy metabolism in CHF rats using various research methods.

**Methods:**

High-performance liquid chromatography (HPLC) analysis was used to perform quality control of SXT preparations. Then, SD rats were randomly assigned into 6 groups: sham, model, positive control (trimetazidine) and high-, middle-, and low-dose SXT groups. Specific reagent kits were used to detect the expression levels of ALT and AST in rats’ serum. Echocardiography was used to evaluate cardiac function. H&E, Masson and TUNEL staining were performed to examine myocardial structure and myocardial apoptosis. Colorimetry was used to determine myocardial ATP levels in experimental rats. Transmission electron microscopy was used to observe the ultrastructure of myocardial mitochondria. ELISA was used to estimate CK, cTnI, and NT-proBNP levels, and LA、FFA、MDA、SOD levels. Finally, Western blotting was used to examine the protein expression of CPT-1, GLUT4, AMPK, p-AMPK, PGC-1α, NRF1, mtTFA and ATP5D in the myocardium.

**Results:**

HPLC showed that our SXT preparation method was feasible. The results of ALT and AST tests indicate that SXT has no side effect on the liver function of rats. Treatment with SXT improved cardiac function and ventricular remodelling and inhibited cardiomyocyte apoptosis and oxidative stress levels induced by CHF. Moreover, CHF caused decrease ATP synthesis, which was accompanied by a reduction in ATP 5D protein levels, damage to mitochondrial structure, abnormal glucose and lipid metabolism, and changes in the expression of PGC-1α related signal pathway proteins, all of which were significantly alleviated by treatment with SXT.

**Conclusion:**

SXT reverses CHF-induced cardiac dysfunction and maintains the integrity of myocardial structure by regulating energy metabolism. The beneficial effect of SXT on energy metabolism may be related to regulating the expression of the PGC-1α signalling pathway.

**Supplementary Information:**

The online version contains supplementary material available at 10.1186/s12906-023-04035-3.

## Introduction

Chronic heart failure (CHF) is a condition in which the heart cannot provide adequate cardiac output to meet the metabolic requirements of the body and regulate venous return [1]. CHF is a major clinical and public health problem with a prevalence of more than 23 million worldwide and is associated with remarkable mortality, morbidity, and healthcare expenditures [2]. Patients with CHF suffer from numerous symptoms that affect their quality of life, including dyspnoea, fatigue, poor exercise tolerance, and fluid retention [3]. Drug treatments can effectively reduce the mortality and readmission rates of patients with CHF, improve quality of life and is the cornerstone of CHF treatment. At present, the cornerstone drug for heart failure has been updated from the original “Golden Triangle” to the “new quadruple” [4], sodium-glucose cotransporter 2 inhibitor (SGLT2i) is another guideline-directed medical therapy drug besides the use of renin-angiotensin system inhibitors (RASi), β-receptor blockers and aldosterone receptor antagonists (MRA). Although these drugs can rapidly improve the hemodynamic metrics of patients, the prognosis and quality of life are still poor [5]. More importantly, long-term use of these chemical drugs may lead to extremely serious side effects, including hypotension and electrolyte depletion [6], and a growing number of patients with CHF have poor response to these drugs [7]. Therefore, there is an urgent need for new therapeutic targets and strategies, especially for the methods aim at various molecular processes involved in CHF with less side effects.

The heart is an organ with high energy demands, in which 60–90% of the energy comes from β-oxidation of fatty acids, with the remaining 10–40% coming from carbohydrate metabolism, including glycolysis, lactate oxidation, and tricarboxylic acid (TCA) [8, 9]. Myocardial energy metabolism dysfunction and energy deficiency are important causes of CHF [10]. In 2004, Van Bilsen-M et al. proposed the concept of metabolic remodelling of the myocardium [11]. Metabolic remodelling is mainly due to the conversion of the cardiac energy metabolism substrate from fatty acid to glucose, which ultimately leads to a lack of high-energy phosphate and a reduction in energy production [12–14]. Metabolic disorders play important roles in the pathogenesis and development of cardiovascular diseases. As the powerhouses of myocardial cells, mitochondria account for about ~ 40% of the volume of myocardial cells and produce about ~ 95% ATP [15, 16]. It can be seen that the structural and functional integrity of mitochondria is crucial for maintaining mitochondrial function and cardiac homeostasis. Peroxisome proliferator-activated receptor γ coactivator 1 α (PGC-1α), which is an important transcriptional coactivator, is not only a key regulator of mitochondrial biosynthesis but also an important factor in metabolism remodelling [17]. PGC-1α is highly expressed in myocardial tissue, which maintains fatty acid oxidation rates and mitochondrial respiratory function [18]. PGC-1α activity can be modulated by AMP-activated protein kinase (AMPK) phosphorylation [19]. Moreover, PGC-1α regulates mitochondrial biogenesis by activating nuclear respiratory factor 1 (NRF-1) and mitochondrial transcription factor A (mtTFA). PGC-1α also participates in a variety of biological processes. In the heart, PGC-1α effectively activates the biological effects of mitochondria and promotes fatty acid oxidation [20, 21]. Some studies have shown that the expression of PGC-1α is decreased in the advanced stage of heart failure accompanied by impairment of mitochondrial number, structure, function. In addition, PGC-1α is an effective defence agent against oxidative stress. Upregulating PGC-1α expression can reduce oxidative stress injury, and downregulating PGC-1α expression directly affects mitochondrial dysfunction [22]. To sum up, regulating cardiac energy metabolism is an effective means to treat CHF.

In recent years, complementary medicine, especially traditional Chinese medicine (TCM), has shown advantages in the treatment of many types of disorders, such as CHF [23]. Shengxian decoction (SXT) is a classic TCM prescription recorded in a practical dictionary of Chinese Medicine by the famous TCM physician Zhang Xi-chun in the late Qing Dynasty and the early Republic of China. Many studies revealed that SXT has good therapeutic effects on the cardiovascular system. It is made up of five kinds of Chinese medicinal materials, including Astragali Radix, Anemarrhenae Rhizoma, Bupleuri Radix, Platycodonis Radix, Cimicifugae Rhizoma. Eighteen constituents were identified in SXT and their types mainly involve, astragaloside IV, ferulic acid, anemarrhena saponin, quercetin, and platycodin D [24]. The results of network pharmacological analysis showed that SXT can reduce the damage of myocardial cells and produce anti-CHF effect mainly through anti-inflammatory, anti-myocardial hypoxia, improving energy metabolism and other pharmacological actions [25]. Research using CHF rat models reveals that SXT had good therapeutic effect on the cardiovascular system [26]. Meanwhile, SXT enjoys obvious therapeutic effect against DOX-induced cardiomyocyte injury in myocardial cells isolated from neonatal rats [27]. Further research shows that SXT can decreases doxorubicin‑induced cardiac apoptosis by regulating the TREM1/NF‑κB signalling pathway [28]. A previous meta-analysis has demonstrated the clinical efficacy of SXT in the treatment of CHF patients, which could significantly improve the left ventricular ejection fraction and the quality of life [29]. However, the molecular mechanisms of SXT in CHF treatment are not well understood. More experimental evidence is still needed to support its effectiveness and safety. Therefore, in the present study, we examined the mechanism of action of SXT in improving CHF in an animal model and focused on the involvement of energy metabolism modulation and related signalling pathways.

## Materials and methods

### Mechanism idea diagram

**Fig. 1:**
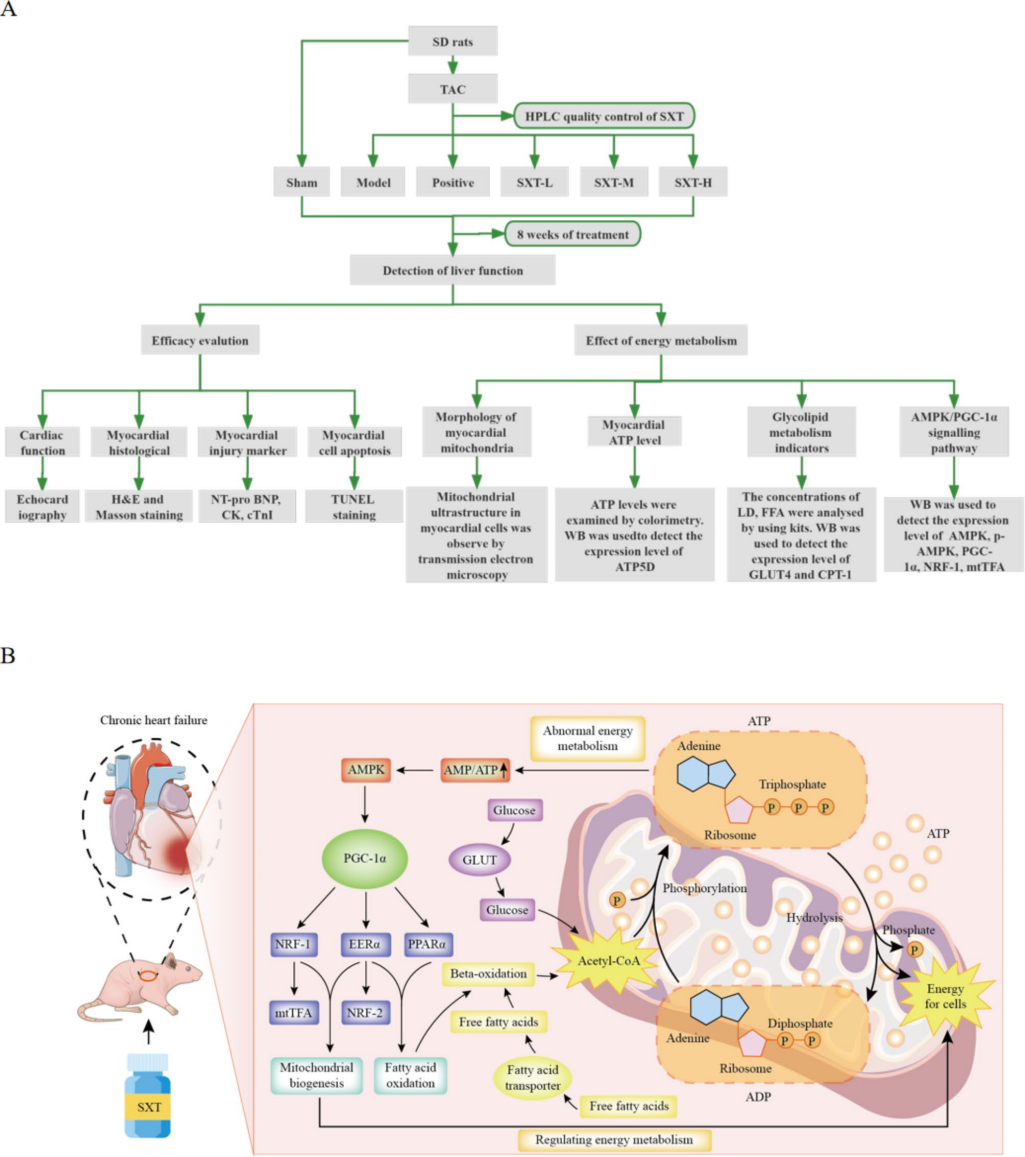
Mechanism idea diagram. **A** Experimental flow chart. **B** Experimental mechanism chart

An overall mechanism idea diagram is shown in Fig. 1.

### Drugs and reagents

Trimetazidine hydrochloride tablets (VASOREL) was purchased from Servier (Tianjin) Pharmaceutical Co., Ltd. (No. 2019118). We prepared SXT using the following dosage: 22.4 g of Astragali Radix, 11.2 g of Anemarrhenae Rhizoma, 5.6 g Bupleuri Radix, 5.6 g Platycodonis Radix, 3.7 g Cimicifugae Rhizom. The medicinal materials were provided by Anhui Boyao Qiancao National Medicines CO., Ltd. High performance liquid chromatography (HPLC) showed that the preparation method of SXT was both stable and feasible.

### High‑performance liquid chromatography (HPLC) analysis of SXT

The quality and chemical constituents of our SXT preparation were examined by HPLC. The condition optimization of the chemical fingerprint was as follows. An Amethyst C18-H (5 μm, 4.6 × 250 mm), with methanol (A) and 0.1% phosphoric acid (B) as the mobile phase was used under full wavelength detection. Gradient elution was used for these solvents. For HPLC analysis, a 20 µl sample was injected into the column, with a column temperature of 30 °C. The detection was performed at 254 nm.

### Animal model and experimental treatments

Male Sprague–Dawley (SD) rats, weighing 150–180 g, were obtained from Liaoning Changsheng Biotechnology Co., Ltd. (Liaoning, China). The rats were maintained in a temperature-controlled environment (24 ± 4 °C) with a 12/12 h light-dark cycle and sufficient food and water. All processes related to animal care and use were carried out in accordance with the guidelines for the Care and Use of Laboratory Animals (NIH Publication No. 85–23, revised 1996 and Ethics Committees in Science: European Perspectives) and were strictly approved by the Institutional Animal Care and Use Committee of Hebei University of Chinese Medicine (DWLL2021039). All possible means were taken to avoid animal suffering at each step in the experiment.

After 7 days of adaptation, the animals were randomly divided into six groups (n = 8): sham group, model group, positive control (trimetazidine) group, high-dose SXT group (SXT-H), middle-dose SXT group (SXT-M), low-dose SXT group (SXT-L). The sham group underwent a similar procedure without ligation. Animals in the other groups were subjected to transverse aortic constriction (TAC) [6]. The rats were anaesthetized by an intraperitoneal injection of 3% pentobarbital sodium at a dose of 1 ml/kg, Then, the rats were fixed in the supine position on the operating table, the hair was removed, an incision was made along the upper edge of the second rib, the mediastinum was opened in layers, the thymus was removed, and the aortic arch at the bottom of the heart was passively separated. A blunt 27-G injection needle (outer diameter 0.4 mm) was placed parallel to the outer wall of the main artery between the brachiocephalic trunk and the left common carotid artery, the needle and the aortic arch were tied together with 5 − 0 surgical silk thread, the needle was removed, the chest was closed, and the operation was finished. Four weeks later, the ligated rats began to receive treatment. All CHF rats in the SXT-L group were treated with 5.1 g/kg/day SXT by intragastric administration. All CHF rats in the SXT-M were treated with 10.2 g/kg/day SXT by intragastric administration. All CHF rats in the SXT-H group were treated with 20.4 g/kg/day SXT by intragastric administration. (The dosage of SXT was approximately one, two, four times the equivalent dose of SXT used in the clinic for adults, which is converted by normalization of body surface area.) In the sham group and model group, the rats were treated with 0.9% physiological saline by intragastric administration. In the positive control group, the rats were treated with 6.3 mg/kg/day trimetazidine by intragastric administration. All groups were administered drugs or normal saline for 8 weeks. After 8 weeks of treatment, the rats were examined by transthoracic echocardiography, and myocardial tissue and blood samples were collected.

### Liver function

The concentrations of alanine aminotransferase (ALT), aspartate aminotransferase (AST) in serum were analysed by using specific kits (Beckman Coulter Laboratory Systems (Suzhou) Co., Ltd., Suzhou, China).

### Echocardiography

The rats were anaesthetized by inhalation of isoflurane (1% oxygen + 5% isoflurane). Transthoracic two-dimensional echocardiography was performed using an MS-250 probe (Vevo 2100, Visual Sonics Inc., Toronto, Canada). The X and Y axes of the console were adjusted to obtain the B-mode long axis section of the parasternal long axis, and the left ventricle and aorta could be seen. The position was unchanged to obtain the M-mode longaxis section ultrasonic image. The sampling line was located at the maximum chamber diameter of the ventricle. Ejection fraction (EF), fractional shortening (FS), left ventricular volume at systole (LVVol; s) and diastole (LVVol; d), left ventricular internal diameter at end-diastole (LVID; d), and left ventricular internal diameter at end-systole (LVID; s) were obtained by long axis measurement (PLAX). Each rat was analysed for 3 cycles, and the average value was taken. All measurements were obtained by an examiner who was blinded to the groupings. After the observation of echocardiography, blood samples were collected for biochemical analysis. The rats were sacrificed by exsanguination following anesthesia, and the tissues were collected for the following experiments [30].

### Serum index

Serum samples were obtained by centrifuging the blood samples at 3500 r·min^− 1^ for 10 min at 15 ℃. The supernatant was collected, immediately stored at -80 ℃, and thawed at -4 ℃ before analysis. The concentrations of creatine kinase (CK), lactic acid (LD), free fatty acid (FFA), malondialdehyde (MDA) and superoxide dismutase (SOD) in serum were analysed by using kits (Nanjing Jian Cheng Biotech Co., Ltd., Nanjing, China). In addition, serum N-terminal pro-B-type natriuretic peptide (NT-pro BNP) levels were analysed using an NT-pro BNP ELISA kit (Sangon Biotech (Shanghai) Co., Ltd., Shanghai, China). Serum cardiac troponin I (cTnI) levels were analyzed using a cTnI ELISA kit (Shanghai Xitang Biotechnology Co., Ltd., Shanghai, China). All analyses were performed in accordance with the manufacturer’s instructions.

### Tissue staining

After 8 weeks of gavage treatment, myocardial tissues were obtained from all rats. Rat heart tissues were fixed in 4% paraformaldehyde for 48 h and then embedded in paraffin. Then, the myocardial tissues were cut into slices with a thickness of 4 μm. Subsequently, the sections were stained with H&E (Nanjing Jian Cheng Biotech Co., Ltd., Nanjing, China) according to the kit protocol. Furthermore, to determine the effect of SXT treatment on cardiac fibrosis, Masson’s trichrome staining was used to stain the tissue sections (Nanjing Jian Cheng Biotech Co., Ltd., Nanjing, China). Collagen tissue appeared blue in colour, and normal tissue was red. The histological changes in the myocardium were observed using a microscope (Olympus Medical Systems Corp, Tokyo, Japan) and analysed.

### Terminal deoxynucleotidyl transferase-mediated deoxyuridine triphosphate nick-end labelling (TUNEL) staining

Cell apoptosis in the myocardial tissues of model rats was analysed by TUNEL assays using an In Situ Cell Death Detection Kit (Solarbio, Beijing, China) according to the manufacturer’s instructions. Specifically, the heart tissues were fixed in 4% paraformaldehyde for 48 h and then embedded in paraffin. Then, 4 μm-thick serial sections were made, deparaffinized, dehydrated in graded alcohol and stained with the reagents in the In Situ Cell Death Detection Kit. Apoptotic nuclei stained by TUNEL were brownish yellow. Finally, the apoptotic cells were observed under a confocal laser scanning microscope.

### Energy metabolism assessment

ATP levels in the myocardium were examined by colorimetry. The tissue was accurately weighed, and 9 times the volume of boiling double-distilled water was added according to the weight (weight (g): volume (ml) = 1:9) to make a 10% homogenate, The homogenate was placed in a boiling water bath for 10 min, removed, mixed well and extracted for 1 min at 3500 RPM. Then, the supernatant was used for testing according to the testing requirements of the specific kit (Nanjing Jian Cheng Biotech Co., Ltd., Nanjing, China).

### Mitochondrial structure

Mitochondrial ultrastructure in myocardial cells was observe by transmission electron microscopy (H-7650, HITACHI, Ibaraki, Japan). After 8 weeks of intragastric treatment, the myocardial tissue of rats was removed, small pieces of myocardial tissue at the apex of the left ventricle were quickly cut into 3 ~ 4 pieces (1 mm × 1 mm × 1 mm), fixed and embedded, and then an ultrathin microtome was used to slice the samples to a thickness of 60 nm. The sections were double stained with 4% uranyl acetate and lead citrate (H-7650, Hitachi, Ibaraki, Japan).

### Western blotting

Total protein was extracted from myocardial tissues by using RIPA lysis buffer (Santa Cruz Biotechnology, Texas, USA). After the protein concentration was determined, equal amounts (20 µg) of protein were mixed with 5 × loading buffer. Then, the mixture was separated on a 12% SDS-PAGE gel and transferred onto a PVDF membrane (Wuhan Servicebio Technology Co., Ltd., Wuhan, China). After that, the membranes were incubated with 5% nonfat milk for 1 h at room temperature. Subsequently, the membranes were incubated with antibody working solutions containing CPT-1 (1:2000, DF12004, Affinity), GLUT4 (1:2000, AF5386, Affinity), AMPK (1:2000, AF6423, Affinity), p-AMPK (1:2000, AF3423, Affinity), PGC-1α (1:2000, AF5395, Affinity), NRF-1 (1:2000, AF5298, Affinity), mtTFA (1:2000, AF0531, Affinity), ATP5D (1:2000, 14,893, ptgcn) at 4 °C overnight. The next day, the membranes were incubated with secondary antibodies for 1 h at room temperature. Finally, the membranes were incubated with ECL reagent (Chengdu ZEN Bioscience Co., Ltd., Chengdu, China) to visualize the protein bands. The relative expression of target proteins was normalized to β-actin. Immunoblot band intensities were quantified using NIH ImageJ software.

### Statistical analysis

SPSS 20.0 software (SPSS Inc., Chicago, IL, USA) was used for statistical analysis. The data are presented as the mean ± standard deviation. Comparisons between two independent groups were determined by Student’s t test. One-way ANOVA was used to analyse significant differences among the three groups. *P* < 0.05 was considered statistically significant. Statistical analysis was performed using GraphPad Prism software (Version 9.0; GraphPad Software, Inc., La Jolla, CA, USA) for Windows.

## Results

### HPLC profile for SXT

**Fig. 2:**
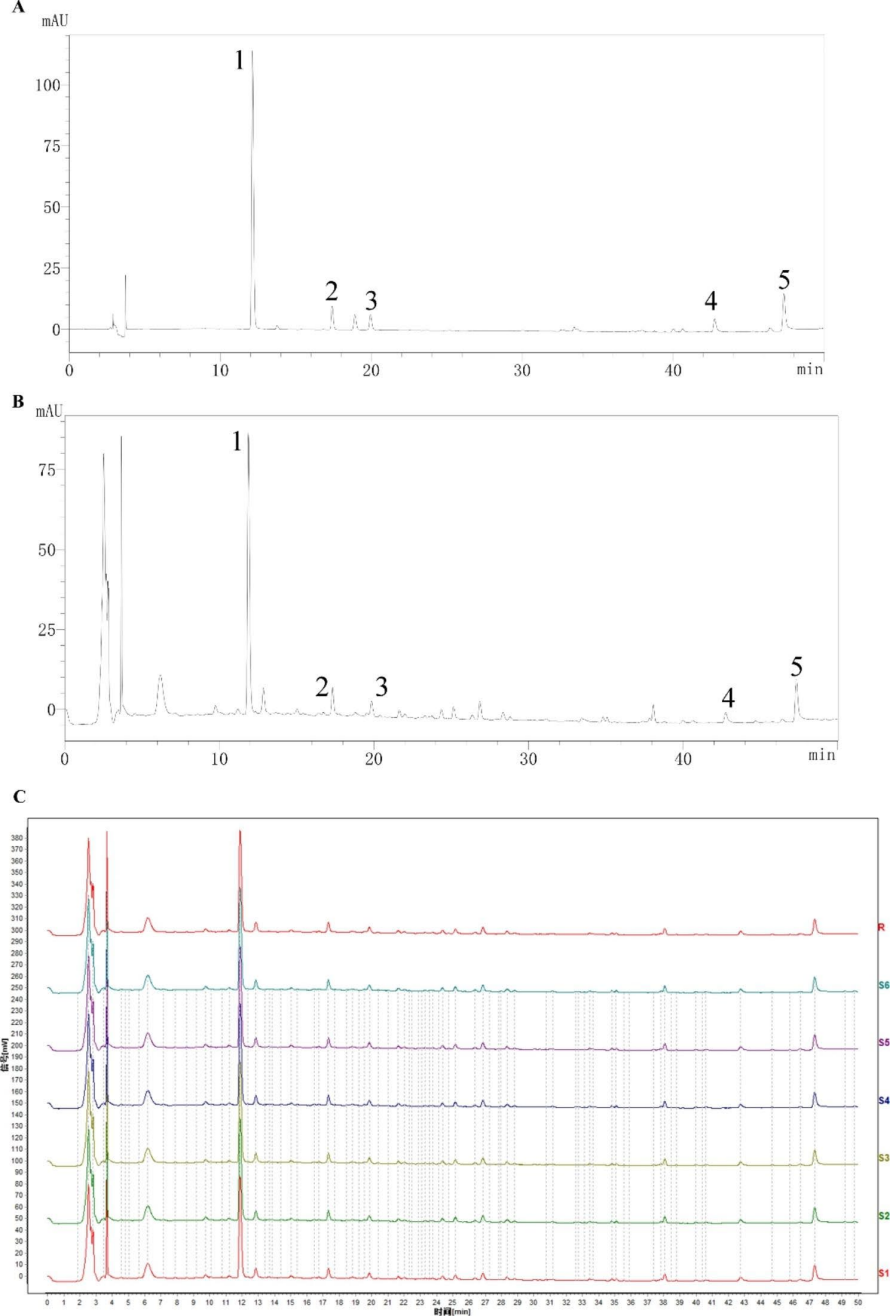
High-performance liquid chromatography (HPLC) chromatogram of a SXT reference sample **(A)** and a test sample **(B)**. From left to right, the index components are (1) mangiferin, (2) ferulic acid, (3) isoferulic acid, (4) calycosin, and (5) saikosaponin a. **C** Fingerprint of SXT (including six batches)

Our previous clinical trials and animal experiments revealed that SXT had a good inhibitory effect on CHF. We also examined the main components of SXT granules to ensure the quality of their preparation. Figure 2 shows the HPLC chromatograms of an SXT reference sample (A) and of a test sample (B). From left to right, the index components are mangiferin, ferulic acid, isoferulic acid, calycosin, and saikosaponin a. The fingerprint results show that the similarity between the samples of each batch and the control samples is greater than 0.96 (Fig. 2C). The results showed that our preparation method for SXT was both stable and feasible, which provides a reference for its quality control.

### Results of liver function

In order to comprehensively evaluate the safety of SXT, we tested the liver function of experimental rats. As shown in Table 1, compared with that of animals in the sham group, the liver function data of rats in the model group changed significantly, which indicates that the model rats have a certain degree of liver injury (Table 1, *P* < 0.01). However, compared with that of animals in the model group, SXT treatment significantly improved liver function (Table 1, *P *< 0.01), which indicates that SXT can prominently alleviate liver dysfunction caused by chronic heart failure, and also indicates that SXT has no side effects of liver injury.

**Table 1:** Liver function test results of rats in each group

Group	ALT(U/L)	AST(U/L)
Sham	31.34 ± 8.68	53.11 ± 7.15
Model	88.92 ± 15.80^**^	114.26 ± 26.11^**^
Positive	38.35 ± 10.08^##^	64.58 ± 17.95^##^
SXT-L	37.68 ± 11.66^##^	63.56 ± 13.40^##^
SXT-M	39.00 ± 9.78^##^	62.40 ± 30.47^##^
SXT-H	34.10 ± 6.62^##^	57.63 ± 9.12^##^

Note: Vs Sham Group, ^*^P < 0.05, ^**^P < 0.01; Vs Model Group, ^#^*P* < 0.05, ^##^*P* < 0.01

### SXT can effectively reduce myocardial injury in CHF rats

**Fig. 3:**
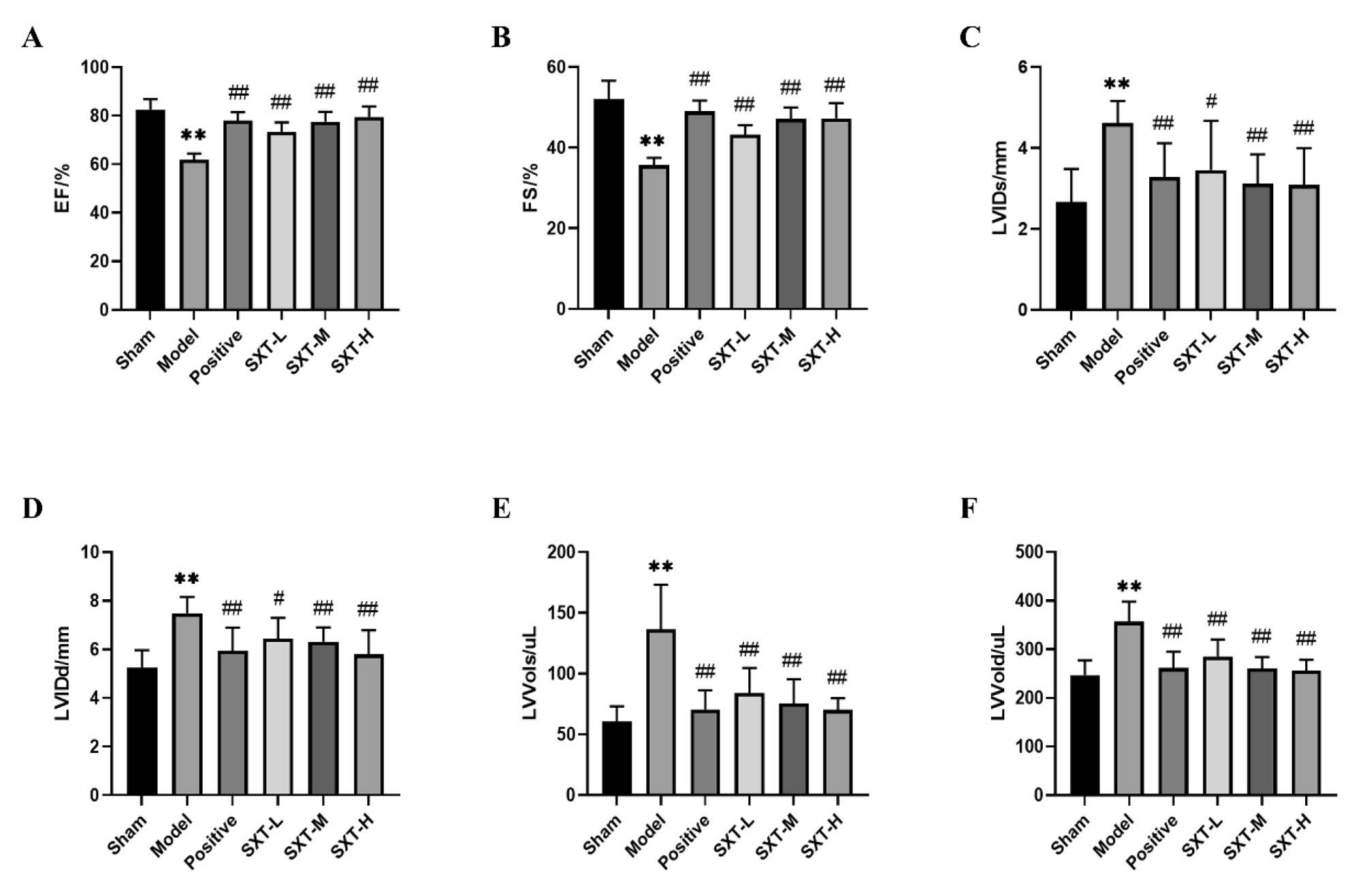
Effect of SXT on the cardiac function of CHF rats. CHF rats were treated with low‑dose (5.1 g/kg/day), middle‑dose (10.2 g/kg/day), and high‑dose (20.4 g/kg/day) SXT. **A** EF in all rats. **B** FS in all rats. **C** LVID; s in all rats. **D** LVID; d in all rats. **E** LVVol; s in all rats. **F** LVVol; d in all rats. ^**^P < 0.01 compared with the sham group. ^#^*P* < 0.05 and ^##^*P* < 0.01 compared with the model group

To study the effect of SXT on cardiac function in CHF rats, we examined the left ventricle ejection fraction (EF), fractional shortening (FS), left ventricular volume at systole (LV Vol; s) and diastole (LV Vol; d), and left ventricular internal systolic (LVID; s) and diastolic (LVID; d) diameters. All animals had normal cardiac function before TAC surgery. As shown in Fig. 3, compared with that of animals in the sham group, the echocardiographic data of rats in the model group changed significantly. TAC rats showed significant cardiac systolic and diastolic dysfunction, EF and FS decreased significantly (Fig. 3A and B, *P* < 0.01), and LVID and LV Vol increased significantly (Fig. 3C-F, *P* < 0.01), indicating that the CHF model was successfully constructed. However, compared with that of animals in the model group, trimetazidine and SXT treatment significantly enhanced cardiac function, significantly increased the levels of EF and FS (Fig. 3A and B, *P* < 0.05 or *P* < 0.01), and significantly reduced LVID and LV Vol (Fig. 3C-F, *P* < 0.05 or* P* < 0.01), suggesting that trimetazidine and SXT could attenuates pathological cardiac dysfunction in a rat model of CHF.

**Fig. 4:**
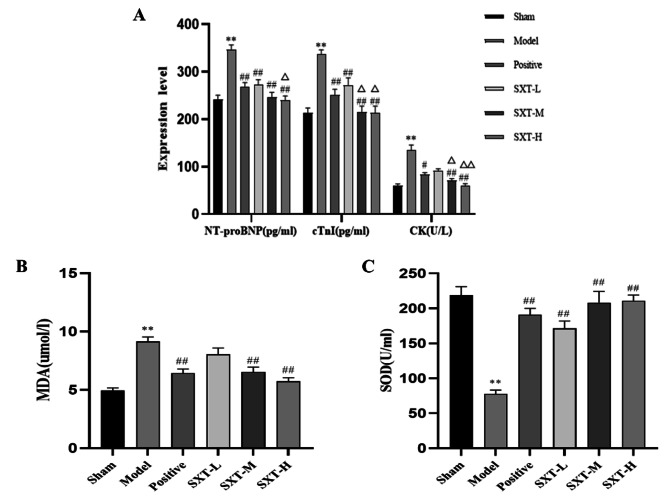
Effect of SXT on the serum myocardial injury indices. **A** Serum concentration of NT-proBNP, cTnI, CK were examined using specific kits. **B** A chemical approach was used to determine the serum concentrations of MDA. **C** A chemical approach was used to determine the serum concentrations of SOD. ^**^P < 0.01 compared with the sham group. ^#^*P* < 0.05 and ^##^*P* < 0.01 compared with the model group. ^#^*P* < 0.05 and ^##^*P *< 0.01 compared with the positive group

To evaluate myocardial damage induced by TAC, serum concentrations of cTnI and CK were analysed in the serum. cTnI and CK were obviously increased in the model group compared with the sham group (Fig. 4A, *P* < 0.01), which indicated myocardial injury in rats. After treatment with different doses of SXT and the positive control drug trimetazidine, serum cTnI and CK levels in each group were improved to varying degrees (Fig. 4A, *P* < 0.05 or *P* < 0.01). Compared with the positive control group, the SXT-M and SXT-H groups had significantly reduced levels of cTnI and CK (Fig. 4A,* P* < 0.05 or* P* < 0.01). It is worth noting that compared with the model group, the SXT-L group had only significantly reduced levels of cTnI and did not show significant changes in the levels of CK. In addition, serum levels of NT-pro BNP were increased significantly in the model group compared to the sham group, which indicated that the risk of CHF was increased (Fig. 4A, *P *< 0.01) [31]. In contrast, CHF rats treated with different dose of SXT showed markedly decreased serum NT-pro BNP levels (Fig. 4A, *P* < 0.01). Compared with the positive control group, the SXT-H group had significantly reduced levels of NT-pro BNP (Fig. 4A, *P *< 0.05). Moreover, MDA and SOD reflect the oxidative stress level of myocardial cells. In the model group, serum MDA levels increased significantly (Fig. 4B, *P* < 0.01), and serum SOD levels decreased significantly (Fig. 4C, *P* < 0.01). Compared with that in the model group, the level of MDA in the SXT-M and SXT-H groups decreased significantly (Fig. 4B,* P* < 0.01) and the serum level of SOD in each SXT treatment group increased significantly (Fig. 4C, *P* < 0.01).

### SXT alleviates myocardial tissue necrosis, myocardial fibrosis and apoptosis

**Fig. 5:**
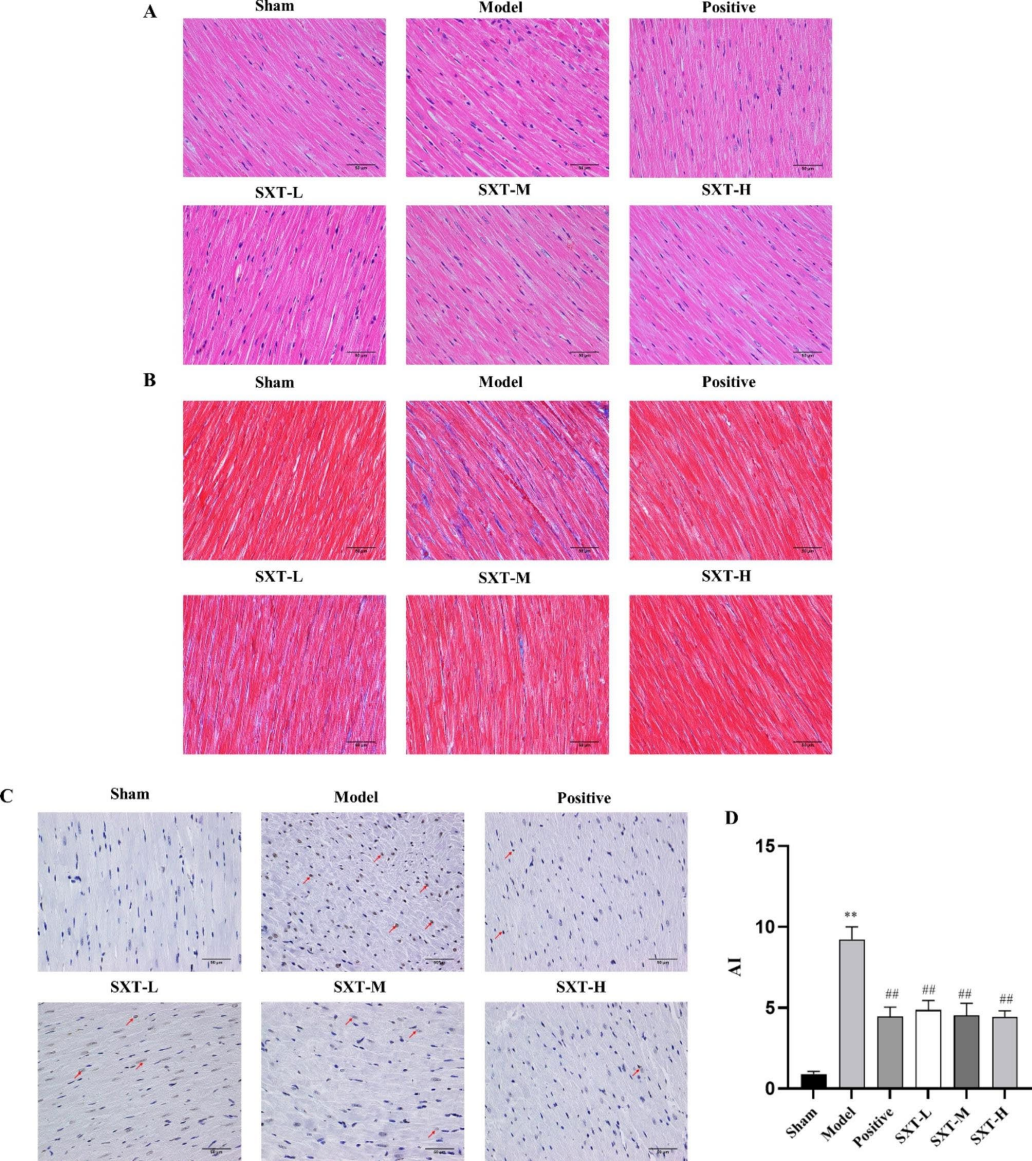
Effect of SXT on myocardial injury in CHF rats. **A** H&E staining was performed to detect pathological changes in the myocardial tissues of rats. **B** Masson staining was performed to detect myocardial fibrosis in rats. **C** TUNEL experiments were performed to detect cell apoptosis in the myocardial tissues of rats (The arrows indicate specific changes). **D** The apoptosis index (AI) in myocardial tissues of rats was analysed. ^**^P < 0.01 compared with the sham group. ^##^*P* < 0.01 compared with the model group

H&E staining showed that the myocardial fibres of rats in the sham operation group were arranged in an orderly and clear manner without obvious deformation. Compared with those in the sham group, myocardial tissues in the CHF model group exhibited broken and necrotic myocardial fibres, and inflammatory cell infiltration could be observed in the necrotic area, while SXT treatment obviously attenuated damage in CHF rats. Notably, this change was particularly evident in the SXT-H group (Fig. 5A).

Masson staining showed that compared with that in sham rats, the myocardial tissue of CHF rats was disorderly arranged, a large number of blue fibrotic tissues could be seen in the myocardial interstitium, and the area of collagen fibres and the degree of myocardial fibrosis were significantly increased. SXT treatment reduced the collagen fibre area, and the myocardial cells were structurally intact and neatly arranged. Similarly, this change was particularly evident in the SXT-H group (Fig. 5B).

In addition, our results showed that the number of apoptotic cells in myocardial tissue of CHF rats was higher than that in normal rats. SXT treatment suppressed apoptosis in the myocardial tissues of CHF rats (Fig. 5C and D).

### SXT enhances the structural and functional integrity of myocardial mitochondria

**Fig. 6:**
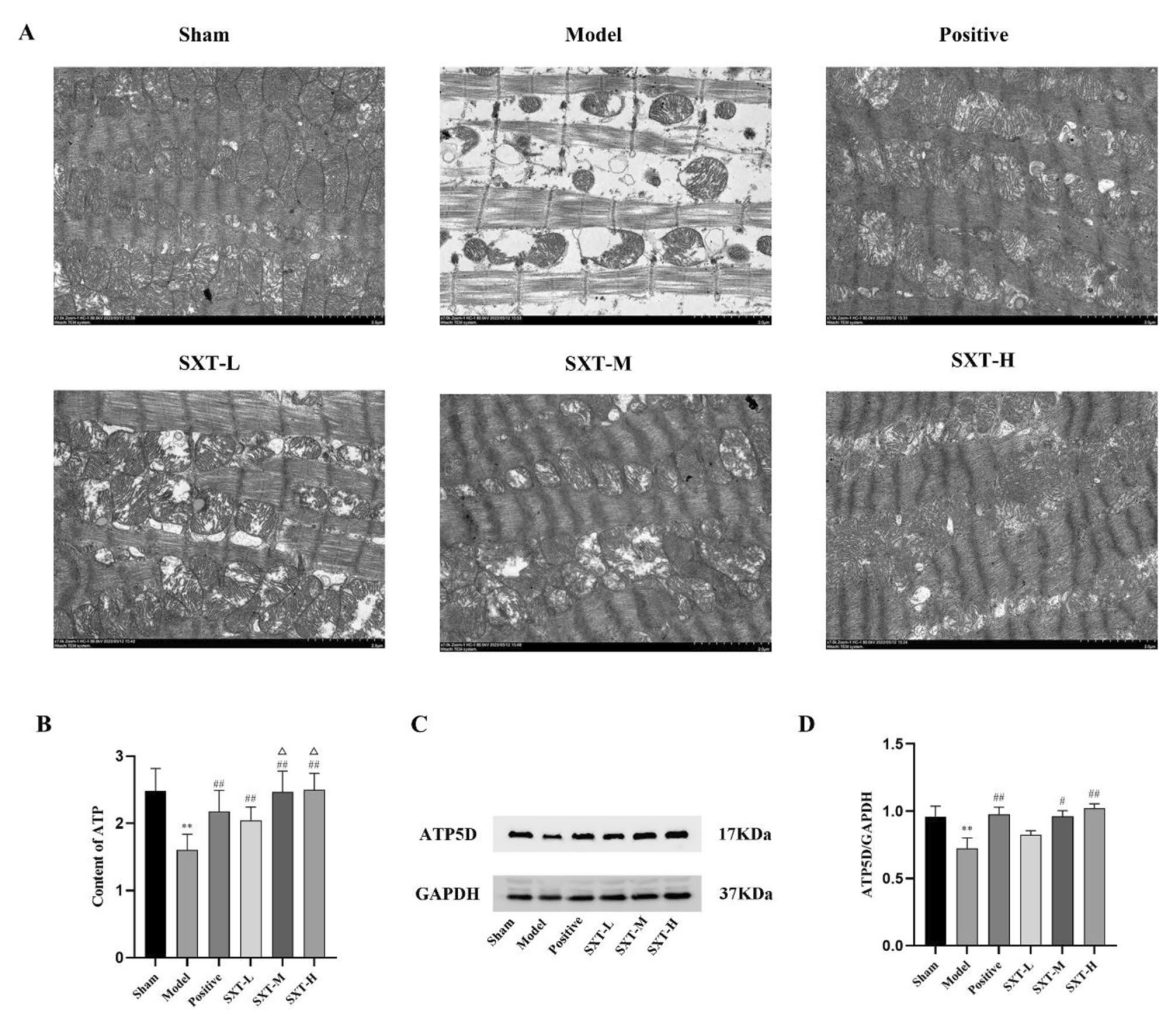
Effect of SXT on the structure and function of myocardial mitochondria in CHF rats. **A** Transmission electron microscopy was used to observe the ultrastructure of myocardial mitochondria. **B** The concentration of ATP in tissue was detected. **C** Western blotting was performed to measure the expression of ATP5D in myocardial tissues. D Quantifcation analysis of ATP 5D and GAPDH. ^**^P < 0.01 compared with the sham group. ^#^*P *< 0.05 and ^##^*P* < 0.01 compared with the model group. $\Delta$ *P* < 0.05 compared with the positive group

We used transmission electron microscopy to observe the ultrastructure of myocardial mitochondria in rats. In the sham group, mitochondria were intact, and myofibrils were regular. However, mitochondria in the model group were fuzzy, integrity was destroyed, and myofibrils were disordered and broken. In response to treatment with SXT, the mitochondria were neatly arranged, there was a reduction in mitochondrial swelling, and the mitochondrial structure became relatively clear and intact (Fig. 6A).

In addition, our results showed that there was no significant change in the ATP levels in myocardial tissue in the sham group. Compared with those in the sham group, ATP levels in the model group decreased significantly (Fig. 6B, *P* < 0.01), suggesting that the hearts of rats with CHF had serious energy deficits. Compared with those in the sham group, ATP levels in the SXT and trimetazidine treatment groups increased significantly (Fig. 6B, *P* < 0.01). Compared with the positive control group, the SXT-M group and SXT-H group had increased ATP levels (Fig. 6B, *P* < 0.05). These results indicate that SXT and trimetazidine can significantly improve energy supply in the hearts of CHF rats, but SXT was more effective. We next determined the expression of ATP 5D in myocardial tissue. The change in ATP levels is related to the expression level of ATP5D, a subunit of ATP synthase. The protein expression of ATP 5D was reduced in CHF rats. Importantly, treatment with SXT-M, SXT-H and trimetazidine ameliorated the alterations induced by CHF (Fig. 6C and D, *P* < 0.05 or *P* < 0.01).

### SXT regulates myocardial energy metabolism pathways

**Fig. 7:**
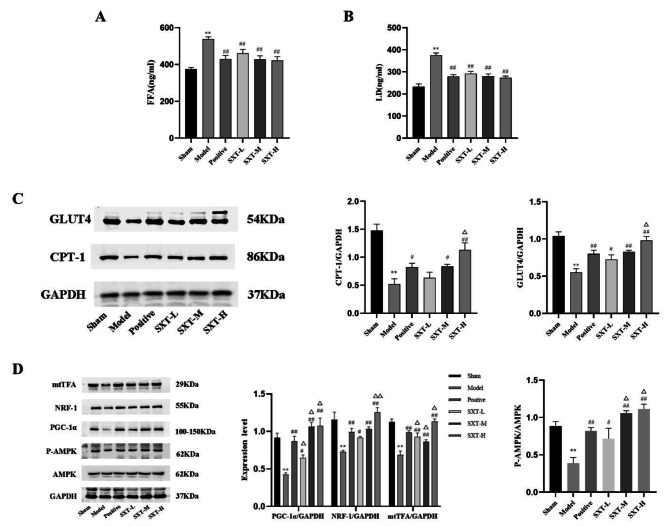
Effect of SXT on myocardial energy metabolism in CHF rats. **A** The concentration of FFAs in the serum of all rats was examined using a specific kit. **B** The concentration of LA in the serum of all rats was examined using a specific kit. **C** Western blotting was performed to measure the expression of GLUT4 and CPT-1 in myocardial tissue. **D** Western blotting was performed to measure the expression of mtTFA, NRF-1, PGC‑1α, p-AMPK and AMPK in myocardial tissue. ^**^P < 0.01 compared with the sham group. ^#^*P* < 0.05 and ^##^*P* < 0.01 compared with the model group. $\Delta$*P* < 0.05 and $\Delta \Delta$*P* < 0.01 compared with the positive group

Our results suggest that SXT may optimize the structure and function of myocardial mitochondria. Therefore, to further examine the effect of SXT on energy metabolism substrates in heart failure, we examined key factors related to glucose and fatty acid metabolism. As shown in Fig. 7A and B, compared with those in the sham group, serum levels of LA and FFA in the model group increased significantly (Fig. 7A and B, *P* < 0.01). Compared with those in the model group, the levels of LA and FFAs in each SXT treatment group and the trimetazidine group decreased (Fig. 7A and B, *P* < 0.01). We also examined the protein expression of CPT- I and GLUT4. Compared with that in the sham group, the protein expression of CPT- I and GLUT4 in the model group was sharply reduced (Fig. 7C, *P* < 0.01). Compared with that in the model group, SXT and trimetazidine treatment obviously improved the protein expression of CPT-I and GLUT4 (Fig. 7C, *P* < 0.05 or* P* < 0.01). In addition, compared with that in the positive control group, the effect in the SXT-H group was more significant (Fig. 7C, *P* < 0.05).

Next, we used Western blotting to measure changes in the protein expression of AMPK, p-AMPK, PGC-1α, NRF1 and mtTFA in the myocardium. Compared with those in the sham group, the protein levels of p-AMPK/AMPK, PGC-1α, NRF1 and mtTFA in the myocardium in the model group decreased significantly (Fig. 7D, *P* < 0.01). Compared with that in the model group, the protein expression of p-AMPK/AMPK, PGC-1α, NRF1 and mtTFA in the myocardium in the SXT groups and positive control group increased significantly (Fig. 7D, *P* < 0.05 or *P* < 0.01). Compared with the positive control group, the SXT-H group exhibited increased expression of all proteins (Fig. 7D, *P* < 0.05 or *P* < 0.01).

## Discussion

CHF, as the final stage of cardiac diseases, is characterized by impaired ventricular filling or ejection due to abnormal structure or function of the heart [32]. The occurrence and development of CHF are complex, and the mechanism is not completely clear. At present, it is generally believed that the core pathological change in heart failure is ventricular remodelling, and the key mechanism is neuroendocrine hormone imbalance, which is characterized by a decline in cardiac function, myocardial hypertrophy, myocardial fibrosis and myocardial cell apoptosis [33]. An important pathological change that causes structural remodelling of the ventricle is metabolic remodelling, which involves changes in the energy metabolism pathway during heart failure and the destruction of mitochondrial function, leading to abnormal changes in the structure and function of the heart. During the process of heart failure, pathological factors such as mitochondrial dysfunction, defects in electron transfer chain activity, the conversion of energy substrate utilization, and energy transport disorders lead to an imbalance in cardiac metabolism, insufficient energy supply in the myocardium, and further damage to cardiac structure and function. It is generally believed that metabolic remodelling occurs earlier than ventricular remodelling. Cardiac structural remodelling and cardiac systolic and diastolic dysfunction are end-point events of energy metabolic remodelling. Therefore, in the current study, we first used HPLC to control the quality of the SXT decoction studied and evaluated the effect of SXT on liver function. Then, we examined whether SXT could improve ventricular structural remodelling in CHF. We found that remarkable cardiac remodelling and cardiac dysfunction occurred in TAC mice, manifested by significantly increased cardiac fibrosis and hypertrophy, as well as diminished echocardiographic features.SXT significantly improved cardiac function, as evidenced by improved EF and FS values. SXT could also significantly improve the myocardial pathological changes in model rats and reduce the degree of myocardial injury and myocardial fibrosis. Our experimental results confirmed that SXT also could decrease serum levels of CK, NT-pro BNP, and cTnI, alleviate oxidative stress levels, and inhibit cell apoptosis. Trimetazidine inhibits fatty acid oxidation by inhibiting the last enzyme in fatty acid oxidation, thus promoting the aerobic oxidation of glucose, reducing oxygen consumption in the heart, and improving energy metabolism in the heart by increasing ATP output. The results of this study show that trimetazidine can prevent and treat heart failure, and improve cardiac function, myocardial fibrosis, myocardial cell apoptosis and other phenotypes of ventricular remodelling in CHF rats, suggesting that energy metabolism therapy can effectively prevent and treat heart failure.

The heart consumes high amounts of energy. More and more evidence show that damaged energy metabolism and mitochondrial function contribute to heart remodeling leading to CHF [34]. Myocardial cells need a large amount of continuous energy to ensure contractile function and the demands of the heart itself. In the event of heart failure, myocardial energy metabolism is greatly affected, resulting in insufficient ATP generation, leaving the heart in a deficient state. Relative and absolute shortages of energy supply to myocardial cells and energy metabolism disorder cause necrosis and fibrosis in myocardial cells, which can exacerbate the degree of heart failure [35]. Studies have shown that the metabolic remodelling of CHF is a kind of overload cardiomyopathy caused by insufficient energy supply or imbalanced glucose and lipid metabolism caused by lack of blood oxygen, and the damage to mitochondrial structure and function is critical, leading to damage to cardiac structure and function. Therefore, we then studied the two aspects of mitochondrial damage and the imbalance in glycolipid metabolism.

Mitochondria are the hub of cell energy metabolism and key factors in cell apoptosis, autophagy and senescence [36]. Mitochondrial dysfunction is one of the important signs of cardiac dysfunction. ATP depletion caused by mitochondrial dysfunction cannot match the energy demand of normal myocardial cells, and the subsequent compensatory changes in the body are the key mechanisms for the development of heart failure. Improving mitochondrial dysfunction is also an important way to prevent and treat myocardial injury [37–39]. We examined the effect of SXT on mitochondrial dysfunction in CHF rats, and we observed the ultrastructure of mitochondria with a transmission electron microscope. Our results confirmed that SXT could significantly improve mitochondrial ultrastructural pathological damage, such as mitochondrial crest fracture, hypertrophy, hyperplasia and severe cavitation, in CHF rats. ATP is the direct energy source for all energy-consuming reactions in the heart. The maintenance of the excitation-contraction coupling physiological function of myocardial cells depends on the calcium pump function of the sarcoplasmic reticulum. The sarcoplasmic reticulum Ca^2+^-ATP enzyme needs enough ATP to absorb Ca^2+^to maintain the contractile properties of myocardial cells. In addition, the change in ATP levels is related to the expression level of ATP5D, a subunit of ATP synthase. As a result, we next examined the level of ATP and the protein expression of ATP 5D in the myocardium. Our results showed that SXT could significantly increase ATP levels and the protein expression of ATP5D in the hearts of rats with CHF. thereby improving the cardiac energy supply during heart failure.

Metabolic substrate utilization is the first step in cardiac energy metabolism. The energy required by the normal heart to maintain systolic function and basic metabolism is mainly provided by ATP produced by fatty acid oxidation and glucose oxidation. Fatty acids are the main metabolic substrates of mitochondrial energy production under physiological conditions, and 60%~90% of energy is from β fatty acids. The remaining 10%~40% is mainly supplied by glucose. In addition, ketone bodies and amino acids can be used as supplementary substrates for myocardial energy metabolism. Myocardial cells can adapt to different pathophysiological conditions by adjusting the utilization ratio and metabolic pathway of different energy metabolism substrates under conditions of increased load, hypoxia, and stress. In the process of cardiac metabolic remodelling, energy metabolism is reprogrammed toward increased utilization of glucose and with significant downregulation of fatty acid oxidation. Even though glucose metabolism consumes less oxygen, it produces fewer amounts of energy compared with fatty acid oxidation, causing the heart to remain in an energy-starved state. We examined changes in glucose metabolism and fatty acid metabolism in rats with CHF. Compared with that in the sham group, the protein expression of glucose oxidation-related GLUT 4 decreased, LA increased, and the expression of the fatty acid oxidation rate-limiting enzyme CPT-I which is related to fatty acid oxidation decreased, while the concentration of FFAs in circulating blood increased. We found that treatment with SXT and trimetazidine could significantly reverse this change. Due to the change in the energy metabolism pathway during heart failure, the energy metabolism of the myocardium switched to embryonic metabolism mode, in which glucose was the main energy source. While the uptake of glucose by cardiac myocytes is increased, their subsequent entry into the mitochondrial is decreased. This leads to a significant reduction in glucose oxidation and an increase in glycolysis [13]. The glucose metabolism disorder parallel to the systolic dysfunction might be partly due to mitochondrial dysfunction or decreased expression of GLUT4 and other proteins involved in glucose oxidation. Another factor that may affect glucose oxidation is that pyruvate may be channeled into anaplerotic pathways [40]. Due to the decrease in oxygen levels, substrate oxidation in myocardial tissue changes, and the utilization of FFAs is significantly reduced in severe heart failure, which reduces the aerobic metabolic efficiency of cardiomyocytes, while the increase in intracellular FFAs concentrations is toxic and aggravates energy metabolism disorders. Furthermore, a severe lack of blood oxygen in severe heart failure can lead to insulin resistance, which reduces the aerobic oxidation of glucose and changes the energy supply mode to glycolysis. Pyruvate is reduced to LA in the hypoxic state, and the increased production of LA aggravates heart exhaustion. On the one hand, the change in substrate metabolism reduces the level of ATP in the myocardium; on the other hand, the increase in LA and the accumulation of FFAs lead to intracellular acidosis and exacerbates myocardial cell injury, which is consistent with the results of this experiment.

AMPK is a cellular energy change receptor that controls lipid and glucose metabolism. When ATP levels decrease, AMPK is rapidly activated. Moreover, AMPK activation may induce a wide range of effects that coordinately improve cardiac function and alleviate metabolic remodeling, thus maintaining the stability of the internal energy environment [6]. Specifically, increased AMPK activity may restore energy supply by stimulating the utilization of fatty acids and glucose [41]. For one thing, AMPK can stimulate fatty acid uptake into mitochondria and subsequent oxidation [42]. Activated AMPK (p-AMPK) further inhibits malonyl-CoA production and activates CPT-1, which controls the rate-limiting step of mitochondrial fatty acid β-oxidation [43]. For another thing, AMPK mediates GLUT4 translocation from cytoplasm to membrane and phosphorylates phosphofructokinase, thus promoting glucose utilization [44]. As a key downstream molecule of AMPK, PGC-1α is a nuclear receptor activator that promotes mitochondrial biosynthesis, regulates mitochondrial quantity and quality, and participates in fatty acid oxidation and thermogenesis. In addition, PGC-1α can regulate the expression of downstream NRF1, TFAM and other mitochondrial function-related factors and participate in maintaining mitochondrial functional stability [17, 45]. It has been confirmed that knocking out PGC-1α in rats could result in a reduction in ATP levels and mitochondrial enzymatic activity, ultimately leading to heart failure [46]. Therefore, to further examine how SXT regulates mitochondrial dysfunction, we analysed changes in the expression of AMPK/p-AMPK, PGC-1α, NRF1 and TFAM pathway factors in each group. SXT significantly increased the protein expression of p-AMPK/AMPK, PGC-1α, NRF1 and TFAM in the myocardium of rats with myocardial injury, especially in the SXT-H group, suggesting that SXT cooperatively mediated the metabolic flexibility of fatty acids and glucose in cardiac energy metabolism, with AMPK/ PGC-1α pathway being the most impacted pathway.

## Conclusion

In conclusion, our data demonstrated that heart failure is caused by abnormal glucose and lipid metabolism, that myocardial mitochondria are critical, and a certain dose of SXT exerts a cardioprotective effect by improving the structure and function of mitochondria and regulating energy metabolic pathways. The potential protective mechanism of SXT may be related to activation of the AMPK/PGC-1α signalling pathway. Our study shows that SXT is a promising and effective treatment and that AMPK/PGC-1α may be a potential target for CHF treatment.

## Electronic supplementary material

Below is the link to the electronic supplementary material.

Additional file 1: Figure S1-S3 Original blot images.

## Data Availability

The datasets used and/or analyzed during the current study are available from the corresponding author on reasonable request.
