# Supplementary material for: Shengxian decoction protects against chronic heart failure in a rat model via energy regulation mechanisms
Source: BMC Complement Med Ther. 2023 Jun 17;23:200. doi: 10.1186/s12906-023-04035-3 (PMC10276516; doi:10.1186/s12906-023-04035-3)
Supplement: Supplementary file 1 — Additional file 1: Figure S1-S3 Original blot images. [file 12906_2023_4035_MOESM1_ESM.pdf]

Supplementary Information

Shengxian decoction protects against chronic heart failure in a rat model via energy regulation mechanisms

Ze-Qi Yang[1†], Yang-Yang Han[1†], Fan Gao[1], Jia-Ye Tian[1], Ran Bai[1], Qiu-Hong Guo[1*], Xing-Chao Liu[1*]

[†]Ze-Qi Yang and Yang-Yang Han are considered to be co-first authors

[*]Correspondence: qiuhong70105@163.com; liuxingchao@hebcm.edu.cn

1 Hebei University of Chinese Medicine, Xinshi South Road No 326, Qiaoxi District, Hebei 050091 Shijiazhuang, China.

Figure 4C.

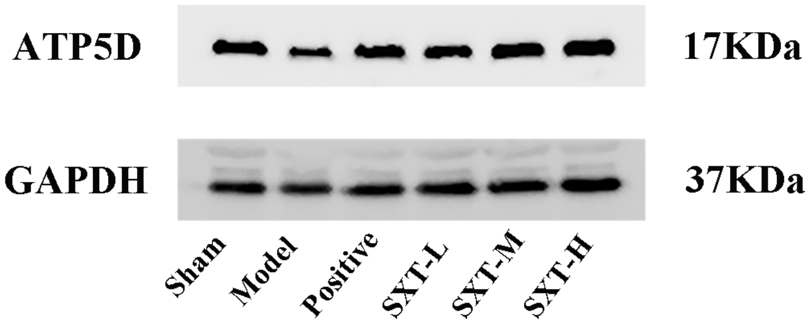

ATP 5D

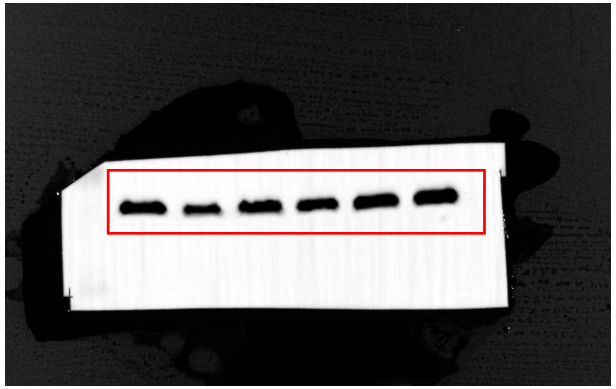

GAPDH

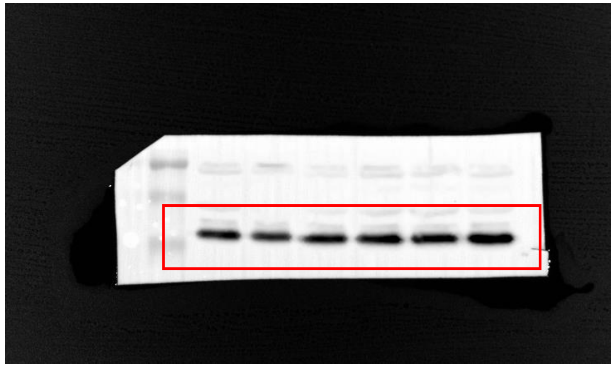

Supplementary Figure S1. The uncropped Western blot images corresponding to Fig.4C showing all the bands

Figure 5C.

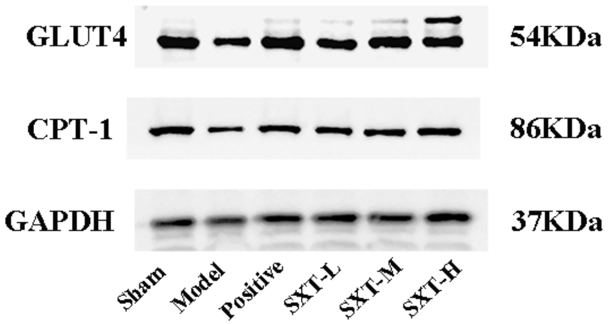

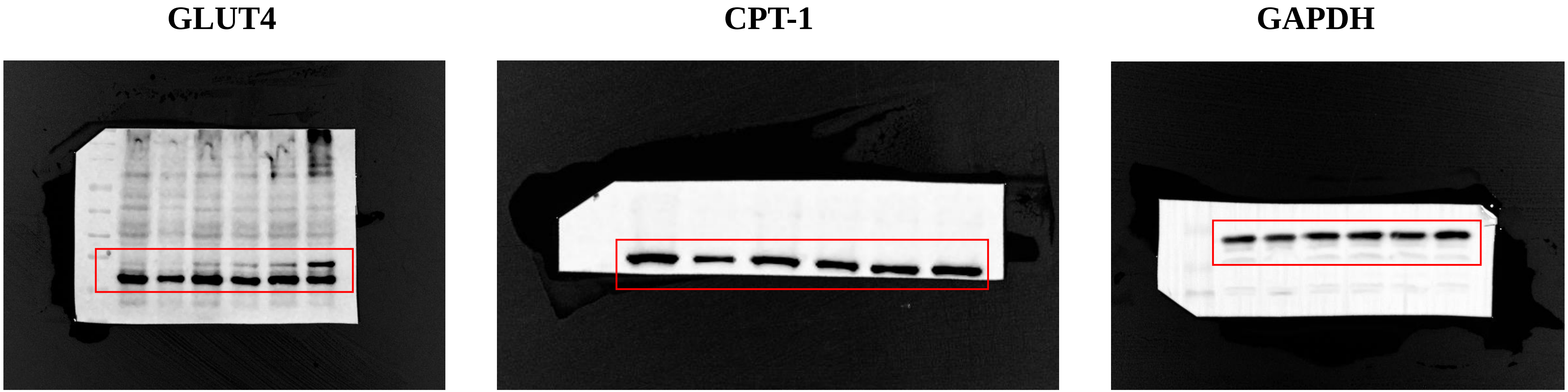

Supplementary Figure S2. The uncropped Western blot images corresponding to Fig.5C showing all the bands

Figure 5D.

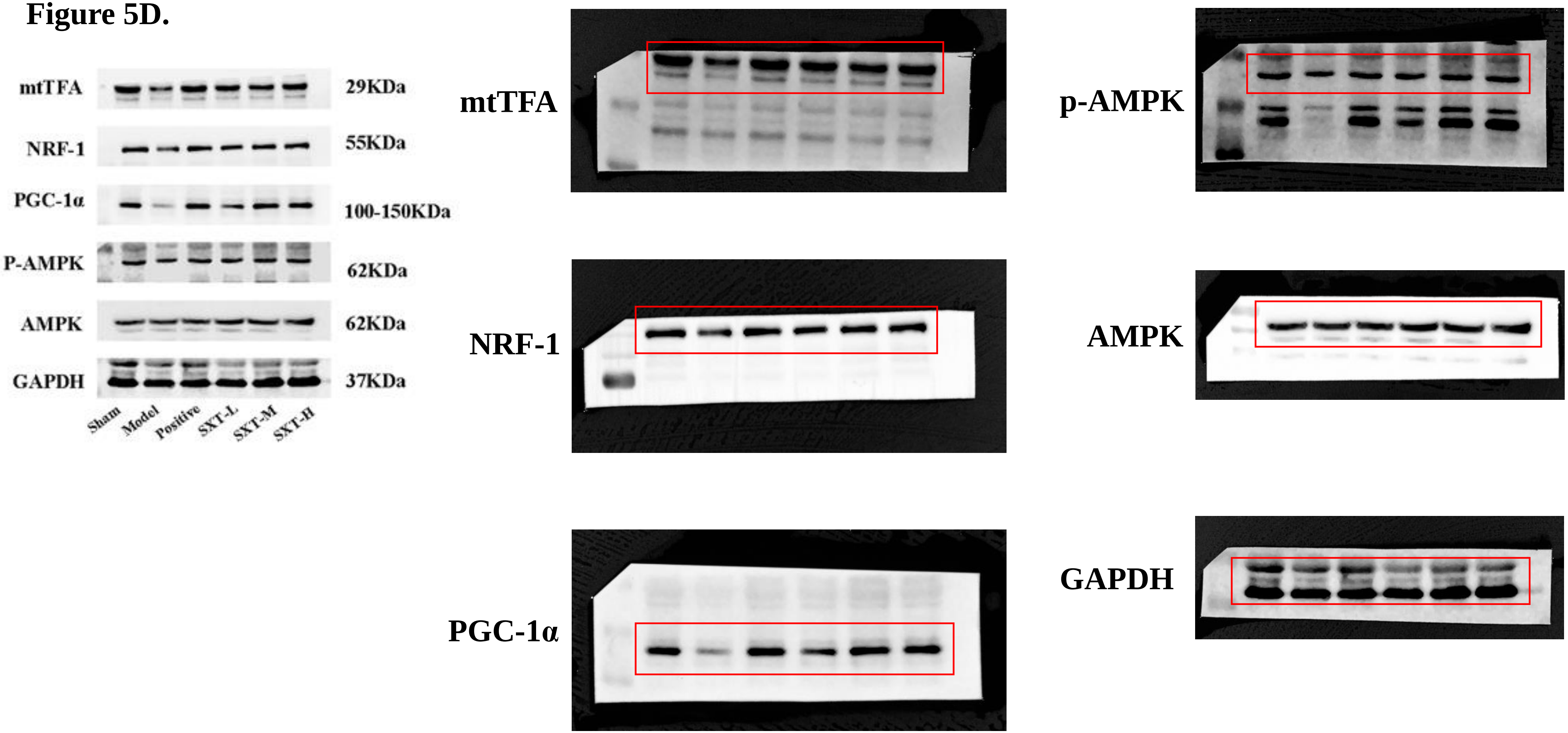

Supplementary Figure S3. The uncropped Western blot images corresponding to Fig.4D showing all the bands
